# Long-Term Antithrombotic Therapy in Patients with Atrial Fibrillation and Percutaneous Coronary Intervention

**DOI:** 10.3390/jcm14113713

**Published:** 2025-05-26

**Authors:** Antonio Capolongo, Vincenzo De Sio, Felice Gragnano, Mattia Galli, Natale Guarnaccia, Pasquale Maddaluna, Giuseppe Verde, Vincenzo Acerbo, Pierre Sabouret, Daniele Giacoppo, Matteo Conte, Silvio Coletta, Vincenzo Diana, Michelangelo Luciani, Elisabetta Moscarella, Arturo Cesaro, Francesco Pelliccia, Paolo Calabrò

**Affiliations:** 1 Department of Translational Medical Sciences, University of Campania “Luigi Vanvitelli”, 80131 Naples, Italy; antonio.capolongo@unicampania.it (A.C.); vincenzo.desio@unicampania.it (V.D.S.); felice.gragnano@unicampania.it (F.G.); natale.guarnaccia@unicampania.it (N.G.); pasquale.maddaluna@unicampania.it (P.M.); giuseppe.verde@unicampania.it (G.V.); vincenzo.acerbo@unicampania.it (V.A.); matteo.conte@aorncaserta.it (M.C.); silvio.coletta@aorncaserta.it (S.C.); vincenzo.diana@aorncaserta.it (V.D.); michelangelo.luciani@unicampania.it (M.L.); elisabetta.moscarella@unicampania.it (E.M.); arturo.cesaro@unicampania.it (A.C.); 2 Division of Cardiology, A.O.R.N. “Sant’Anna e San Sebastiano”, 81100 Caserta, Italy; 3 Department of Medical-Surgical Sciences and Biotechnologies, Sapienza University of Rome, 04100 Latina, Italy; mattia.galli@uniroma1.it; 4 Maria Cecilia Hospital, GVM Care & Research, 48033 Cotignola, Italy; 5 Heart Institute, Cardiology Department, Pitié-Salpétrère Hospital, Sorbonne University, 75005 Paris, France; pierre.sabouret@action-coeur.org; 6 Division of Cardiology, Azienda Ospedaliero-Universitaria Policlinico “G. Rodolico-San Marco”, University of Catania, 95124 Catania, Italy; daniele.giacoppo@unict.it; 7 Department of Cardiovascular Sciences, Sapienza University, 00185 Rome, Italy; francesco.pelliccia@uniroma1.it

**Keywords:** antithrombotic therapy, P2Y_12_ inhibitors, atrial fibrillation, PCI, bleeding

## Abstract

The optimal long-term antithrombotic treatment of patients with atrial fibrillation (AF) undergoing percutaneous coronary intervention (PCI) remains controversial. Current guidelines recommend a short initial period of triple antithrombotic therapy (e.g., 1 week), followed by dual therapy consisting of an oral anticoagulation agent and a single antiplatelet agent for 6 months in patients undergoing elective PCI and 12 months in patients with acute coronary syndromes. After this course of combination therapy, anticoagulation monotherapy is recommended. In daily practice, however, the optimal strategy for long-term antithrombotic therapy remains debated. A growing body of evidence supports the safety and efficacy of oral anticoagulation monotherapy, but its use in clinical practice remains inconsistent. This review aims to evaluate the available evidence on chronic antithrombotic regimens in patients with AF undergoing PCI, with a focus on key clinical considerations, such as the selection of optimal long-term therapy that balances ischemic and bleeding risks. It also highlights that, despite robust supporting evidence, significant gaps persist in real-world implementation.

## 1. Introduction

Atrial fibrillation (AF) is the most prevalent cardiac arrhythmia and correlates with a significantly increased risk of stroke and systemic embolism [1]. Oral anticoagulation (OAC) is the cornerstone of therapy for preventing thromboembolic events in this setting [2].

AF frequently coexists with coronary artery disease, particularly in older patients with multiple cardiovascular risk factors. Notably, 20–30% of patients with AF will require percutaneous coronary intervention (PCI), while approximately 12% of patients undergoing PCI have concurrent chronic or new-onset AF, necessitating long-term OAC [3,4,5,6]. In these patients, a combination of antiplatelet and anticoagulant therapy is required during the first year after PCI, with specific regimens tailored to the clinical presentation [7]. At 6 months for patients with chronic coronary syndrome (CCS) or 12 months for those with acute coronary syndromes (ACS), guideline recommendations suggest transitioning to OAC monotherapy (Class I) [2].

Nevertheless, the optimal timing for stopping antiplatelet therapy in patients with AF undergoing PCI remains controversial. Although randomized controlled trials have provided important evidence, extrapolation of their results to long-term antithrombotic strategies in real-world practice is still inconsistent and often influenced by individual patient risk profiles and physician preferences. Bleeding risk scores may help identify vulnerable patients and modifiable risk factors (such as uncontrolled hypertension or concomitant use of medications that increase bleeding risk), therefore supporting individualized treatment decisions. However, the direct applicability of traditional risk scores like HAS-BLED [8] to the AF-PCI population is limited, as these tools were not specifically developed or validated in this clinical context. More recently, scores such as the PRECISE-HBR [9] have been developed and validated as potentially more appropriate instruments to improve bleeding risk stratification and inform clinical management in patients with AF undergoing PCI. In this review, we examine this issue and summarize the available evidence on long-term antithrombotic strategies in patients with AF and remote PCI.

## 2. Antithrombotic Therapy in AF-PCI Patients

The management of antithrombotic therapy in AF patients undergoing PCI represents a significant challenge, as it requires striking a balance between preventing recurrent coronary events, mitigating the risk of cardioembolic complications, and limiting the risk of bleeding [9,10,11].

In the early post-PCI phase, triple antithrombotic therapy (TAT)—including dual antiplatelet therapy (DAPT) with aspirin and clopidogrel plus OAC—is recommended to address both atherothrombotic and cardioembolic risks [2]. However, TAT is associated with an elevated risk of bleeding, with studies indicating that concomitant use of anticoagulants and antiplatelet agents can increase bleeding complications by up to fivefold [12,13].

The recommended duration of TAT is determined by the individual bleeding risk, with guidelines advising its limitation to the first week post-PCI [2]. In patients with a high ischemic risk—where stent thrombosis risk exceeds bleeding concerns—TAT may be extended up to 1 month (class of recommendation IIa, level of evidence C) [2]. Beyond this period, and up to 12 months in the setting of ACS or 6 months in CCS, aspirin is typically discontinued while OAC is continued in combination with a single antiplatelet agent.

For long-term management beyond 12 months post-PCI, both European and American guidelines recommend OAC monotherapy [2,14]. This recommendation is based on evidence derived from studies conducted in previous decades, where warfarin monotherapy proved non-inferior to aspirin for secondary prevention of myocardial infarction [15]. Recent evidence consistently indicates that long-term dual antithrombotic therapy should be avoided due to an unfavorable risk–benefit ratio, with a chronic direct oral anticoagulants (DOACs) monotherapy to be preferred over vitamin K antagonists (VKAs) in this setting given their improved outcomes in the AF population [16]. Many cardiologists remain reluctant to discontinue antiplatelet therapy after PCI, mainly because of the concern about late stent thrombosis [17,18], even if it has been shown that the actual risk of late stent thrombosis (>12 months) is extremely low in the current era of PCI, especially with the use of newer generation drug-eluting stents [19]. As a result, a considerable proportion of AF patients undergoing PCI receive long-term OAC in combination with single antiplatelet therapy (SAPT), thus diverging from current guideline recommendations and contradicting current evidence [2,20].

## 3. Observational and Randomized Studies of Long-Term Antithrombotic Regimens in AF-PCI

In recent years, observational and randomized studies have increasingly explored the efficacy and safety of OAC monotherapy as an alternative to dual antithrombotic therapy (DAT) in patients with stable coronary artery disease (CAD) and/or remote PCI (>6 months) [21,22,23,24,25,26,27,28,29,30]. This approach aims to minimize bleeding risk while maintaining sufficient thromboembolic protection. Although these investigations have provided important insights, the evidence from individual studies remains limited and often lacks the statistical power necessary to reach definitive conclusions. An important factor that may influence the interpretation and generalizability of the reported outcomes across different studies is the heterogeneity in follow-up durations, as shown in Table 1 (for example a 3.3-year follow-up in the Lamberts’ study [24] vs. a 12-month follow-up in the AFIRE [27]), as longer or shorter observation periods can substantially impact the incidence rates of both ischemic and bleeding events.

### 3.1. OAC-ALONE Trial

**Figure 1:**
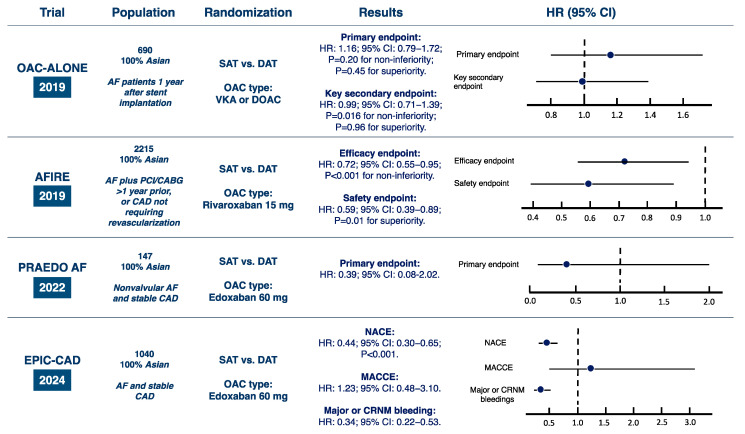
Design and main results of the randomized controlled trials in AF-PCI patients. AF, atrial fibrillation; CAD, coronary artery disease; CI, confidence interval; CRNM, clinically relevant nonmajor; DAT, dual antithrombotic therapy; DOAC, direct oral anticoagulant; HR, hazard ratio; MACCE, major adverse cardiac and cerebrovascular events; NACE, net clinical adverse events; OAC, oral anticoagulation; PCI, percutaneous coronary intervention; SAT, single antithrombotic therapy; VKAs, vitamin K antagonists.

The OAC-ALONE (Optimizing Antithrombotic Care in Patients With Atrial Fibrillation and Coronary Stent) [23] trial was the first prospective, multicenter, randomized, non-inferiority trial designed to compare OAC monotherapy with DAT in patients with AF 1 year after coronary stent implantation. Both VKAs, with international normalized ratio (INR) targets of 2.0–3.0 for patients younger than 70 years and 1.6–2.6 for those aged 70 years or older, and DOACs were permitted. Yet, in both the OAC-only and DAT arms, approximately three-quarters of patients received VKAs. Aspirin or clopidogrel were allowed as antiplatelet agents, although most participants received aspirin (Table 1). The primary end point was a composite of all-cause death, myocardial infarction, stroke, or systemic embolism. The major secondary end point was a composite of the primary end point or major bleeding according to the International Society on Thrombosis and Haemostasis classification. Over 38 months, 696 patients were enrolled, which fell substantially below the planned target of 2000 patients. Indeed, due to slow recruitment, the trial was terminated prematurely, resulting in insufficient power to demonstrate the non-inferiority of OAC monotherapy for the primary endpoint, which occurred in 15.7% of the OAC-only group and 13.6% of the DAT group (HR: 1.16; 95% CI: 0.79–1.72; *p* = 0.20 for non-inferiority; *p* = 0.45 for superiority). The key secondary endpoint occurred in 19.5% of patients in the OAC monotherapy group and 19.4% in the DAT group (HR: 0.99; 95% CI: 0.71–1.39; *p* = 0.016 for non-inferiority; *p* = 0.96 for superiority), thus potentially indicating non-inferiority over a median follow-up of 2.5 years (Figure 1).

Although the study was underpowered and inconclusive in rejecting the null hypothesis, analysis of individual outcomes revealed that myocardial infarction and stent thrombosis were more frequent in patients who did not receive antiplatelet therapy (myocardial infarction incidence: 2.3% in the OAC monotherapy group vs. 1.2% in the DAT group; stent thrombosis incidence: 0.58% in the OAC monotherapy group vs. 0% in the DAT group). Conversely, hemorrhagic stroke (1.2% vs. 1.7%) and major bleeding (7.8% vs. 10.4%) were more prevalent in the DAT group; the incidence of ischemic stroke was identical between the two arms (3.5% vs. 3.5%).

### 3.2. AFIRE Trial

The AFIRE (Atrial Fibrillation and Ischemic Events with Rivaroxaban in Patients with Stable Coronary Artery Disease) trial [24] was a multicenter study conducted in Japan. This study included 2236 patients with AF who had undergone PCI or coronary artery bypass grafting (CABG) performed over a year earlier or who had angiographically documented CAD not requiring revascularization.

Patients were randomly assigned to receive rivaroxaban monotherapy (15 mg/day or 10 mg/day) or a combined regimen of rivaroxaban with one antiplatelet agent. The primary efficacy endpoint was a composite of stroke, systemic embolism, myocardial infarction, unstable angina requiring revascularization, or all-cause mortality, analyzed for non-inferiority. The primary safety endpoint was major bleeding, as defined by the ISTH criteria, analyzed for superiority (Table 1).

The trial was terminated early due to an excess mortality observed in the combination therapy group. For the primary efficacy endpoint, rivaroxaban monotherapy resulted in a significantly lower event rate (4.14% per patient-year) compared to combination therapy (5.75% per patient-year) (HR: 0.72; 95% CI: 0.55–0.95; *p* < 0.001 for non-inferiority). For the primary safety endpoint, rivaroxaban monotherapy demonstrated a lower risk of major bleeding (1.62% per patient-year) compared to combination therapy (2.76% per patient-year) (HR: 0.59; 95% CI: 0.39–0.89; *p* = 0.01 for superiority) (Figure 1).

Despite its significant findings, the study had several limitations. It was conducted exclusively in Japan, limiting generalizability to other populations, as East Asian patients may exhibit distinct pathophysiological characteristics in AF, CAD, and bleeding risk. Additionally, the Japan-approved dose of rivaroxaban (10–15 mg/day) differs from the globally approved regimen, potentially impacting the extrapolation of results to broader clinical practice.

Based on data from the AFIRE trial, Noda et al. [25] conducted a sub-analysis to evaluate whether the efficacy and safety of rivaroxaban monotherapy were consistent in patients with and without prior coronary revascularization (Table 1). Among the 2215 patients included in the modified intention-to-treat population, 1697 (76.6%) had undergone coronary revascularization more than 1 year before enrollment, comprising 1445 patients who had undergone PCI and 252 patients who had undergone CABG. The remaining 518 patients (23.4%) had angiographically confirmed CAD not requiring revascularization. In patients with prior revascularization, rivaroxaban monotherapy resulted in a notable decrease in ischemic events compared to combination therapy (HR: 0.62; 95% CI: 0.45–0.85; *p* = 0.003). In contrast, no significant benefit was observed in patients without prior revascularization (HR: 1.19; 95% CI: 0.67–2.12; *p* = 0.554), suggesting potential heterogeneity in the treatment effect (*p* value of interaction = 0.055). The relative risk of major bleeding was approximately halved with rivaroxaban monotherapy, regardless of prior revascularization status (in patients with prior revascularization, monotherapy 1.76% vs. combination therapy 2.85%, with HR: 0.62; 95% CI: 0.39–0.98; *p* = 0.633), while the increased mortality associated with combination therapy was more pronounced in patients with prior revascularization. The benefits of rivaroxaban monotherapy were consistent across both the PCI and CABG subgroups. Overall, these findings suggest that rivaroxaban monotherapy may offer greater benefits in patients with a history of coronary revascularization, reducing both ischemic and bleeding risks. However, its role in patients with CAD who have not undergone revascularization remains less well defined, highlighting the need for further research [25].

### 3.3. PRAEDO AF Trial

The multicenter PRAEDO AF (Prospective Randomized Assessment of Edoxaban Monotherapy for Patients with Nonvalvular Atrial Fibrillation and Stable Coronary Artery Disease) trial [26] evaluated the safety of edoxaban monotherapy in patients with nonvalvular AF and stable CAD, including those who had received a third-generation drug-eluting stent for at least 6 months or another stent type for at least 1 year. The study enrolled 147 patients, who were randomized into two groups: edoxaban monotherapy (*n* = 74) and edoxaban plus clopidogrel (*n* = 73) (Table 1). The primary endpoint was the incidence of major or clinically significant bleeding according to the ISTH criteria. Bleeding events were lower in the monotherapy group (1.67% per patient-year) than in the combination therapy group (4.28% per patient-year), with an HR of 0.39 (95% CI: 0.08–2.02). No ischemic events, myocardial infarction, stent thrombosis, unstable angina or stroke were reported in either group (Figure 1). These results suggest that edoxaban monotherapy offers acceptable clinical safety in patients with AF and stable CAD. The study’s limitations include a lower-than-expected patient enrollment due to slow entry and the COVID-19 pandemic, underpowered statistical analysis due to fewer bleeding events than anticipated, potential bias from the open-label design, variations in edoxaban dosage and pre-enrollment P2Y_12_ inhibitor use, and differences in DES systems among patients.

### 3.4. MASTER DAPT Trial Sub-Analysis

The MASTER DAPT (Management of High Bleeding Risk Patients Post Bioresorbable Polymer Coated Stent Implantation with an Abbreviated versus Standard DAPT Regimen) trial [30] evaluated the optimal duration of DAPT in patients at high risk of bleeding after sirolimus-eluting stent implantation [30]. A sub-analysis of the MASTER DAPT trial [29] specifically evaluated the subgroup of patients with an indication for OAC therapy. In patients with OAC (*n* = 1666), the abbreviated strategy included an immediate switch to SAPT for 5 months, while the standard regimen included at least another 2 months of DAPT followed by SAPT. In patients without OAC (*n* = 2913), SAPT lasted for 11 months in the abbreviated group, while the standard group continued DAPT for at least another 5 months before switching to SAPT. Three co-primary outcomes were assessed: net adverse clinical events, defined as the composite of all-cause death, myocardial infarction, stroke, and BARC type 3 or 5 bleeding; major adverse cardiac and cerebrovascular events (MACCE), comprising all-cause death, myocardial infarction, and stroke; and major or clinically relevant non-major bleeding, defined as BARC type 2, 3, or 5 bleeding events. At 335 days, there were no significant differences between the abbreviated and standard strategies in terms of net clinical adverse events (HR: 0.83; 95% CI: 0.60–1.15 in patients with OAC, and HR: 1.01; 95% CI: 0.77–1.33 in patients without OAC) and major cardiac and cerebrovascular adverse events (HR: 0.88; 95% CI: 0.60–1.30 with OAC, and HR: 1.06; 95% CI: 0.79–1.44 without OAC). The rate of MCRB did not differ between the two treatment groups in patients on OAC (HR: 0.83; 95% CI: 0.62–1.12), but it was lower in the abbreviated antiplatelet group compared to the standard group in patients without OAC (HR: 0.55; 95% CI: 0.41–0.74) (Figure 1). A key limitation of this sub-analysis is that it does not primarily address long-term antithrombotic therapy in AF-PCI patients, as it also includes the early post-PCI phase. In addition, although the MASTER DAPT trial included stratified randomization for the presence of an indication to OAC, the analysis of this subgroup remains a secondary analysis of a randomized controlled trial, and should therefore be interpreted with caution. In particular, the results may be underpowered for specific endpoints and are subject to the inherent limitations of subgroup analyses, despite the preservation of randomization within the OAC stratum.

### 3.5. EPCIC-CAD Trial

The EPIC-CAD [28] was a multicenter randomized trial designed to compare edoxaban monotherapy with DAT (edoxaban plus a single antiplatelet agent) in patients with AF and stable CAD. A total of 1040 patients were enrolled, with 524 assigned to the edoxaban monotherapy group and 516 to the DAT group. At 12 months, the primary endpoint (defined as a composite of death from any cause, myocardial infarction, stroke, systemic embolism, unplanned urgent revascularization, and major bleeding or clinically relevant nonmajor bleeding) occurred in 34 patients (6.8%) in the edoxaban monotherapy group compared to 79 patients (16.2%) in the DAT group (HR: 0.44; 95% CI: 0.30–0.65; *p* < 0.001), indicating a significant reduction in adverse outcomes with edoxaban monotherapy. The cumulative incidence of major ischemic events at 12 months was similar between the two groups. The incidence of clinically relevant major or non-major bleeding was significantly lower in the edoxaban monotherapy group (23 patients, 4.7%) compared with the dual therapy group (70 patients, 14.2%) (HR: 0.34; 95% CI: 0.22–0.53) (Figure 1).

The EPIC-CAD trial demonstrated that edoxaban monotherapy was associated with a significantly lower risk of a composite endpoint, including fatal and non-fatal ischemic and bleeding outcomes, compared to DAT in patients with AF and stable CAD, suggesting that long-term OAC monotherapy in this patient population can reduce bleeding risk without compromising ischemic protection (Table 1) [28].

### 3.6. Observational Studies

In 2014, a large observational study from the nationwide Danish administrative registries, [21] involving 8700 patients (mean follow-up of 3.3 years) investigated the effect of VKA therapy alone or combined with antiplatelet agents. Compared with VKA monotherapy, the addition of aspirin (HR: 1.12; 95% CI: 0.94–1.34) or clopidogrel (HR: 1.53; 95% CI: 0.93–2.52) did not reduce the incidence of myocardial infarction or coronary death. Similarly, thromboembolic risk remained comparable among all VKA regimens, but bleeding risk significantly increased when aspirin (HR: 1.50; 95% CI: 1.23–1.82) or clopidogrel (HR: 1.84; 95% CI: 1.11–3.06) was added to the VKA therapy. These findings suggest that, in patients with AF and stable CAD, combining VKA with antiplatelet therapy confers no additional benefit in preventing coronary events or thromboembolism, while substantially elevating bleeding risk (Table 1).

In 2022, using data from the West Danish Cardiac Registry, Jensen et al. examined outcomes in AF patients who underwent their first PCI between 2003 and 2017 [22]. The study compared 3331 patients who received monotherapy vs. DAT, and 1275 patients who received DOACs vs. VKAs as a monotherapy. The risk of hospitalization for bleeding (HR: 0.90; 95% CI: 0.75–1.09) and major adverse cardiovascular events (MACE) (HR: 1.04; 95% CI: 0.90–1.19) was similar between the monotherapy and DAT groups. Moreover, the risk of hospitalization for bleeding (HR: 1.27; 95% CI: 0.84–1.92) and MACE (HR: 1.15; 95% CI: 0.87–1.50) did not differ significantly between DOAC and VKA monotherapy. These findings from real-world registry cohorts support the adoption of long-term OAC monotherapy beyond 1 year following PCI in patients with AF, indicating that DOAC monotherapy is safe and effective in this population (Table 1) [22].

## 4. Synthesis of the Available Evidence from Meta-Analyses

Several meta-analyses have recently been conducted to synthesize the available evidence in this setting. Montalto et al. [31] conducted a meta-analysis to evaluate the optimal duration of DAPT in patients requiring concomitant OAC. The study included data from five randomized controlled trials, enrolling a total of 7665 patients, of whom 3843 received an abbreviated DAPT regimen, while 3822 were treated with an extended DAPT strategy (≥3 months). The co-primary efficacy endpoints were MCRB and major bleeding, while the composite endpoint of MACE was designated as the key safety endpoint. The findings demonstrated that an abbreviated DAPT regimen significantly reduced both MCRB (risk ratio (RR): 0.69; 95% CI: 0.52–0.91; *p* = 0.01) and major bleeding (RR: 0.70; 95% CI: 0.52–0.95; *p* = 0.01). No significant differences were observed between the two groups in terms of MACE (RR: 0.96; 95% CI: 0.70–1.33; *p* = 0.60) or in the incidence of all-cause mortality, cardiovascular mortality, stent thrombosis, or myocardial infarction. Furthermore, a network meta-analysis indicated that a periprocedural DAPT regimen exhibited the highest probability of reducing MCRB (97.1%) and major bleeding (92.0%) when compared with both shorter (4–6 weeks) and longer (≥3 months) DAPT durations. Sensitivity analyses and meta-regressions reinforced these findings, suggesting that the bleeding risk reduction following DAPT discontinuation was more pronounced with P2Y_12_ inhibitors than with aspirin.

Another meta-analysis conducted by Ahmed et al. [32] evaluated data from three randomized trials involving 3945 patients with AF and stable CAD, comparing OAC monotherapy with DAT for clinical outcomes. The results indicated that OAC monotherapy was associated with a significantly lower risk of major bleeding compared to DAT (RR: 0.55; 95% CI: 0.32–0.95). In contrast, no significant differences were observed between the two treatment strategies in terms of all-cause mortality (RR: 0.85; 95% CI: 0.49–1.48), cardiovascular mortality (RR: 0.84; 95% CI: 0.50–1.41), any type of stroke (RR: 0.74; 95% CI: 0.46–1.18), or myocardial infarction (RR: 1.57; 95% CI: 0.79–3.12). This meta-analysis confirmed that OAC monotherapy significantly reduces the risk of major bleeding while maintaining comparable rates of ischemic events and mortality relative to DAT.

A more recent and comprehensive meta-analysis [33] further assessed the efficacy and safety of OAC monotherapy compared to OAC plus SAPT in patients with AF and stable CAD, incorporating all currently available randomized evidence. This study included 4092 patients with a median follow-up of 21.9 months, drawing data from four randomized controlled trials. The primary efficacy endpoint, defined as a composite of myocardial infarction, ischemic stroke, systemic embolism, or all-cause mortality, did not differ significantly between OAC monotherapy and OAC plus SAPT (7.3% vs. 8.2%; HR: 0.90; 95% CI: 0.72–1.12). Similarly, no significant differences were observed for individual endpoints, including myocardial infarction (1.0% vs. 0.7%; HR: 1.51; 95% CI: 0.75–3.04), ischemic stroke (1.9% vs. 2.1%; HR: 0.89; 95% CI: 0.57–1.37), all-cause mortality (4.2% vs. 5.3%; HR: 0.94; 95% CI: 0.49–1.80), and cardiovascular mortality (2.4% vs. 3.0%; HR: 0.79; 95% CI: 0.54–1.15). Importantly, OAC monotherapy was associated with a significantly lower risk of major bleeding compared to OAC plus SAPT (3.3% vs. 5.7%; HR: 0.59; 95% CI: 0.44–0.79). Subgroup analyses suggested that the magnitude of bleeding reduction was more pronounced in male patients (P interaction = 0.03) and in those with diabetes mellitus (P of interaction = 0.04).

Collectively, these meta-analyses reinforce the role of OAC monotherapy in patients with AF and stable CAD, demonstrating that it preserves ischemic protection while significantly reducing the risk of major bleeding (Table 2) [34,35].

## 5. Practical Recommendations by Current Guidelines

**Figure 2:**
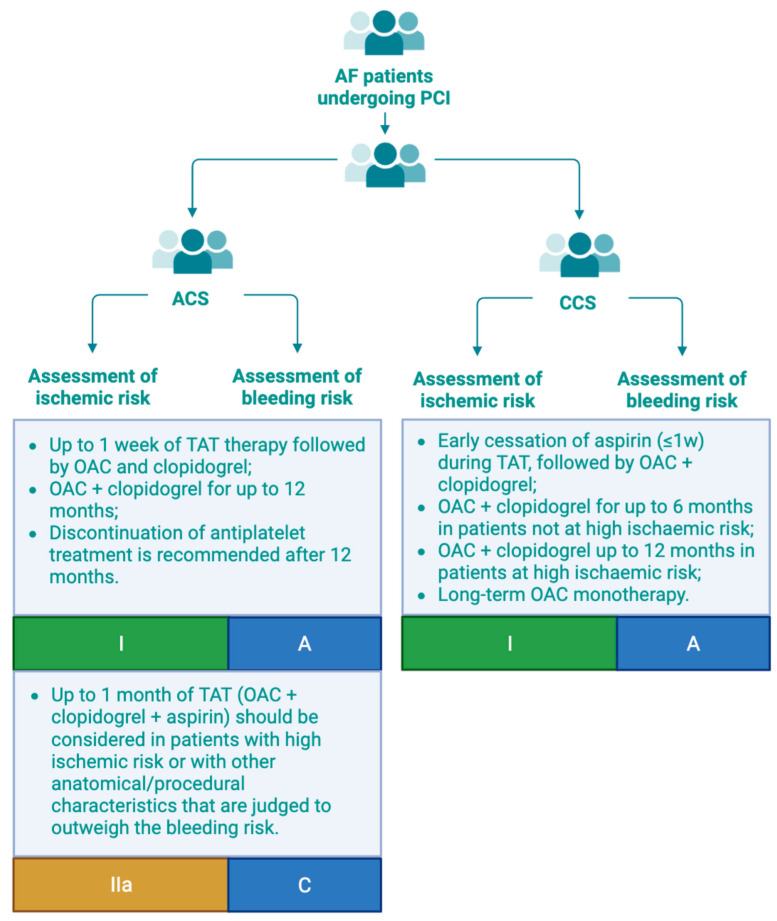
Practical recommendations by current guidelines for patients with AF undergoing PCI. AF, atrial fibrillation; ACS, acute coronary syndrome; CCS, chronic coronary syndrome; OAC, oral anticoagulation; PCI, percutaneous coronary intervention; TAT, triple antithrombotic therapy.

Based on the current evidence, current European guidelines [2] recommend an initial period of 1 week of TAT, followed by 12 months of a P2Y_12_ inhibitor, preferably clopidogrel, in combination with OAC for patients with AF and ACS undergoing uncomplicated PCI, provided the risk of thrombosis is low and the bleeding risk is high (class of recommendation I, level of evidence A). In patients where the ischemic risk outweighs the bleeding risk, TAT may be extended up to 1 month (class of recommendation IIa, level of evidence C). For patients with AF undergoing elective PCI, a strategy of 1 week of triple therapy, followed by 6 months of DAT if the ischemic risk is low (class of recommendation I, level of evidence A). If the risk of stent thrombosis exceeds the risk of bleeding, triple therapy can be prolonged for up to 1 month [2]. To minimize the risk of bleeding and improve outcomes, antiplatelet therapy should not be extended beyond 12 months, irrespective of clinical presentation (class of recommendation III, level of evidence B) (Figure 2) [2].

The American guidelines [14] underscore that recent evidence from randomized clinical trials suggests that OAC monotherapy, particularly rivaroxaban, may be preferable to a combination therapy in patients with AF who have stable CAD and no history of stent thrombosis in order to reduce the risk of major bleeding, with a class I recommendation and B-R level of evidence.

## 6. Conclusions

Long-term anticoagulant therapy in patients with AF who have undergone PCI remains a clinical challenge that requires an individualized approach to balance risks and benefits. Stroke prevention in these patients is essential, and the risks of recurrent coronary events and stent thrombosis have to be minimized. At the same time, the bleeding risk associated with long-term OAC must be considered, especially when anticoagulation is combined with antiplatelet therapy. Overall, the available evidence indicates that, beyond the first year after PCI, OAC monotherapy (preferably with a DOAC) is the best long-term regimen for AF patients with stable CAD, given its favourable efficacy and safety in preventing thromboembolic events and reducing bleeding complications.

## Figures and Tables

**Table 1:** Studies comparing long-term single versus dual antithrombotic regimens in AF-PCI patients.

Study	Year	Sample Size	Design	CHA_2_DS_2_-VASc Score (Median)	HAS-BLED Score (Median)	Monotherapy Regimen	Dual Therapy Regimen	Endpoints	Follow-Up (Median)
OAC-ALONE [23]	2019	696	RCT	4.6	2	Warfarin (75.2%) or DOACs (24.8%)	Warfarin or DOACs plus aspirin or clopidogrel	Primary endpoint: death, MI, stroke, or SE. Key secondary endpoint: primary endpoint or major bleeding (ISTH criteria).	30 mo.
AFIRE [24,25]	2019	2236	RCT	4	2	Rivaroxaban 15 mg once daily	Rivaroxaban 15 mg once daily plus aspirin or a P2Y_12_ inhibitor	Efficacy endpoint: stroke, systemic embolism, MI, unstable angina requiring revascularization, or death. Safety endpoint: major bleeding (ISTH criteria).	24.1 mo.
PRAEDO AF [26]	2022	147	RCT	4	3	Edoxaban 60 mg once daily	Edoxaban 60 mg once daily plus clopidogrel	Primary endpoint: major bleeding or clinically significant bleeding (ISTH criteria).	20.8 mo.
EPIC-CAD [28]	2024	1040	RCT	4	2.2	Edoxaban 60 mg once daily	Edoxaban 60 mg once daily plus aspirin or a P2Y_12_ inhibitor	NACE: death, MI, stroke, systemic embolism, unplanned urgent revascularization, major or CRNM bleeding (ISTH criteria). MACCE: death, MI, stroke, or SE. Major or CRNM bleeding.	12 mo.

CRNM, clinically relevant non-major; DOAC, direct oral anticoagulant; ISTH, International Society on Thrombosis and Haemostasis; MACCE, major adverse cardiac and cerebrovascular events; MI, myocardial infarction; mo., months; NACE, net clinical adverse events; PCI, percutaneous coronary intervention; RCT, randomized clinical trial; SE, systemic embolism.

**Table 2:** Meta-analyses evaluating long-term single- versus dual-antithrombotic regimens in AF-PCI patients.

Meta-Analysis	Year	Sample Size	No. of RCTs Included	RCTs Included	Arms	Endpoints and Results
Montalto et al. [31]	2023	7665	5	WOEST trial; ISAR-TRIPLE trial; AUGUSTUS trial; SAFE-A trial; MASTER DAPT trial.	Abbreviated DAPT regimen vs. extended DAPT strategy (≥3 mo.)	MCRB: RR: 0.69; 95% CI: 0.52–0.91; *p* = 0.01 Major bleeding: RR: 0.70; 95% CI: 0.52–0.95; *p* = 0.01) MACE: RR: 0.96; 95% CI: 0.70–1.33; *p* = 0.60
Ahmed et al. [34]	2024	3945	3	AFIRE; OAC-ALONE; EPIC-CAD.	OAC monotherapy vs. DAT	Major bleeding: RR: 0.55; 95% CI: 0.32–0.95 All-cause mortality: RR: 0.85; 95% CI: 0.49–1.48 CV mortality: RR: 0.84; 95% CI: 0.50–1.41 Stroke: RR: 0.74; 95% CI: 0.46–1.18 MI: RR: 1.57; 95% CI: 0.79–3.12
Rashedi et al. [35]	2025	4092	4	AFIRE; OAC-ALONE; PRAEDO AF; EPIC-CAD.	OAC monotherapy compared to OAC plus SAPT in patients with AF and stable CAD	PE (composite of MI, ischemic stroke, SE, or all-cause mortality): 7.3% vs. 8.2%; HR: 0.90; 95% CI: 0.72–1.12 MI: 1.0% vs. 0.7%; HR: 1.51; 95% CI: 0.75–3.04 Ischemic stroke: 1.9% vs. 2.1%; HR: 0.89; 95% CI: 0.57–1.37 All-cause mortality: 4.2% vs. 5.3%; HR: 0.94; 95% CI: 0.49–1.80 CV mortality: 2.4% vs. 3.0%; HR: 0.79; 95% CI: 0.54–1.15 Major bleeding: 3.3% vs. 5.7%; HR: 0.59; 95% CI: 0.44–0.79

AF, atrial fibrillation; CAD, coronary artery disease; CI, confidence interval; CV, cardiovascular; DAPT, dual antiplatelet therapy; DAT, dual antithrombotic therapy; HR, hazard ratio; MACE, major adverse clinical events; MCRB, major or clinically relevant non-major bleedings; MI, myocardial infarction; mo., months; OAC, oral anticoagulant; PE, primary endpoint; RR, risk ratio; SAPT, single antiplatelet therapy.
